# Effectiveness of low dose cyproterone acetate compared to standard dose in feminizing hormone treatment: a single institutional retrospective pilot study

**DOI:** 10.1093/sexmed/qfae063

**Published:** 2024-09-21

**Authors:** Sira Korpaisarn, Jiraporn Arunakul, Kewalin Chaisuksombat, Teerapong Rattananukrom

**Affiliations:** Department of Medicine, Faculty of Medicine Ramathibodi Hospital, Mahidol University, Ratchathewi, Bangkok 10400, Thailand; Department of Pediatric, Faculty of Medicine Ramathibodi Hospital, Mahidol University, Ratchathewi, Bangkok 10400, Thailand; Department of Pediatric, Faculty of Medicine Ramathibodi Hospital, Mahidol University, Ratchathewi, Bangkok 10400, Thailand; Department of Medicine, Faculty of Medicine Ramathibodi Hospital, Mahidol University, Ratchathewi, Bangkok 10400, Thailand

**Keywords:** transgender, gender-affirming hormone therapy, transfeminine, cyproterone acetate, anti-androgens

## Abstract

**Background:**

Data regarding the effectiveness of low-dose cyproterone acetate (CPA) in testosterone suppression as feminizing hormone therapy (FHT) in individuals assigned male at birth (AMAB) are sparse.

**Aim:**

To assess the effectiveness in testosterone suppression using low-dose CPA (<25 mg/day) compared to standard-dose CPA (25–50 mg/day) in FHT.

**Methods:**

A retrospective cohort study of 59 individuals AMAB using CPA was done at a tertiary care center in Bangkok, Thailand between January 2014 and July 2022.

**Outcomes:**

The main outcomes included a median time when the testosterone was suppressed (<50 ng/dL), the proportion of individuals AMAB who achieved the targeted testosterone level at 3 months, and the testosterone level at each follow-up. Changes in clinical data were assessed.

**Results:**

Among 59 individuals AMAB, 37 initiated CPA with available testosterone levels at the 3-month follow-up. Twenty-two individuals AMAB started with low-dose CPA (12.5 mg/day), and 15 individuals AMAB started with standard-dose CPA. The median time to reach targeted testosterone was 3 months in both groups (adjusted hazard ratio 0.60, *P* = .489). At 3 months, 72.7% of those on low-dose CPA and 86.7% of those on standard-dose CPA achieved targeted testosterone (adjusted relative risk 0.85, *P* = .606). Testosterone levels at all follow-up visits were not significantly different. The standard dose group had higher high-density lipoprotein cholesterol (HDL-C) but lower low-density lipoprotein cholesterol (LDL-C) and alanine aminotransferase (ALT).

**Clinical Translation:**

This study supports a paradigm shift toward using lower-dose CPA in FHT.

**Strengths and Limitations:**

This is one of a few studies showing the effectiveness of low-dose CPA in testosterone suppression within 3 months. Limitations include a small sample size and missing data.

**Conclusions:**

Testosterone suppression is comparable between CPA 12.5 mg/day and the standard dose in FHT.

## Introduction

Gender incongruence refers to transgender and gender diverse (TGD) individuals with gender identities or expressions that are markedly and persistently incongruent with the gender socially attributed to the sex assigned at birth.^1^^,^^2^ Although gender diversity is not pathological and is considered a variation in human beings, gender incongruence may cause gender dysphoria, which is significant distress and discomfort associated with being TGD.^3^ TGD individuals may seek gender-affirming treatment to align their physical appearance with their gender identity. Many TGD individuals assigned male at birth (AMAB) pursue feminizing gender-affirming medical and/or surgical treatment. Feminizing gender-affirming hormone therapy (FHT) aims to induce feminine secondary sexual characteristics through a hormone regimen. This is achieved by maintaining estradiol and testosterone levels in the premenopausal cisgender women’s range, which is 100–200 pg/mL for estradiol and less than 50 ng/dL for testosterone.^2^

The common FHT for individuals AMAB who have not undergone gonadectomy includes estrogens and antiandrogens. The latter is unnecessary if they have completed a gonadectomy.^1^^,^^2^ The advantages of adding antiandrogens include the effective suppression of testosterone levels within the target range, allowing for a lower dose of estrogen. Commonly prescribed antiandrogens include spironolactone, cyproterone acetate (CPA), and gonadotropin-releasing hormone (GnRH) agonists, without a specific preference from the guidelines.^1^^,^^2^ The decision on which to use depends on underlying medical conditions, availability, affordability, and physicians’ preferences. Spironolactone is commonly used in the USA, while the National Health Service in the United Kingdom provides GnRH agonists.^4^^,^^5^ CPA is the most common antiandrogen used in European countries as well as in Thailand.^4^^,^^6^

CPA is a progestogenic compound with anti-androgenic effects. Its mechanisms of action include the suppression of testosterone production through its antigonadotropic effect, increased testosterone inactivation by an enzymatic inducer effect on the liver, inhibition of 5alpha-reductase activity, and competitive blocking of the androgen receptor. The end results are both reducing the testosterone level and blocking testosterone’s action.^7^ The United States FDA has disapproved CPA due to a risk of severe hepatotoxicity. However, CPA is commonly used outside the USA. Adverse effects of CPA, apart from hepatotoxicity, include venous thromboembolism, hyperprolactinemia, changes in lipid levels, and meningioma.^1^^,^^8–11^ CPA has been recommended as an alternative treatment for prostate cancer at doses of 100–300 mg/day.^12^ CPA is also an option among other antiandrogens for the additional treatment of hirsutism that has not improved after 6 or more months of combined oral contraceptives, at doses of 50–100 mg/menstrual cycle, from days 5 to 15.^13^

The 2017 Endocrine Society guidelines recommend a CPA dose of 25–50 mg/day for FHT.^2^ The dose should be adjusted to keep the testosterone level below 50 ng/dL, although doses up to 100 mg/day have been mentioned.^14^ The European Society for Sexual Medicine released a position statement in 2020 recommending CPA 10–50 mg/day.^15^ The most recent standard of care version 8 (SOC8), launched by the World Professional Association for Transgender Health (WPATH) in 2022, recommends a CPA of less than 10 mg/day due to a risk of meningioma at higher doses.^1^ This recommendation has changed from the previous standard of care version 7 in 2012, which recommended a CPA dose of 25–50 mg/day.^16^ Although the WPATH officially recommended the lower dose of CPA in 2022, this practice has already been used in Europe^15^ and Thailand for some time. A cross-sectional survey from our group revealed that the CPA dose of 12.5 mg/day was the most commonly reported in real-world practice.^6^ In Thailand, only a 50 mg-dosage form is available, and individuals AMAB typically use a quarter of a tablet, approximately 12.5 mg, as their FHT. However, data regarding the effectiveness of a lower dose of CPA are limited.

In this study, we aimed to evaluate the effectiveness of testosterone suppression using a lower dose of CPA. The goal was to answer the question: Is low-dose CPA (<25 mg/day) effective in suppressing testosterone compared to the standard dose of CPA, 25–50 mg/day? We hypothesized that testosterone suppression would be comparable between the 2 groups.

## Materials and methods

### Study population

This study was a single-institution retrospective cohort study of individuals AMAB who received FHT at the Gender Variation (Gen V) clinic, a gender-affirming clinic at the Faculty of Medicine Ramathibodi Hospital, Mahidol University in Bangkok, Thailand. The Gen V clinic was founded in January 2014 specifically for gender-affirming care for all TGD individuals. Data were collected from all individuals AMAB who continued care between January 2014 and July 2022. We included individuals AMAB who were over the age of 16 during the study period and used CPA as an antiandrogen in their FHT.

Individuals AMAB using CPA 25–50 mg/day were classified as the standard-dose group according to the 2017 Endocrine Society guidelines,^2^ and individuals AMAB using CPA less than 25 mg/day were classified as the low-dose group. The present study has been carried out in accordance with The Code of Ethics of the World Medical Association (Declaration of Helsinki) for experiments involving human subjects. It was approved by the Institutional Review Board of the Faculty of Medicine Ramathibodi Hospital (approval number COA. MURA2022/534), and written informed consent was exempted.

### Feminizing hormone regimen

The FHT consisted of an estradiol agent in combination with CPA for individuals AMAB who had not undergone gonadectomy. Estradiol agents were either oral estradiol (2–6 mg/day) or transdermal 0.06% 17-beta estradiol gel (1.5–6.0 mg/day). The 1.5 mg (2.5 g of gel) transdermal 0.06% 17-beta estradiol gel is equivalent to the estradiol transdermal patch 0.05 mg/day,^17^ which is within the recommended dose according to the guidelines.^1^^,^^2^ Neither injectable estradiol nor estrogen patches were available at our institute. The only dosage form of CPA available is 50 mg/tablet. The initial CPA doses were decided based on the physician’s preference and discussion with individuals AMAB. Follow-up visits were every 3 months in the first year and then every 6–12 months. At the Gen V clinic, the treatment protocol generally follows the 2017 Endocrine Society Clinical Practice Guideline: Endocrine Treatment of Gender-Dysphoric/Gender-Incongruent Persons,^2^ except for the CPA dose, which could be lower than the recommended dose in some individuals AMAB.

### Data collection

Electronic medical records were reviewed, and clinical data, including demographic information, gender history, age of FHT initiation, age of CPA initiation, underlying medical conditions, height, and feminizing hormone regimen, were assessed at baseline when the CPA was initiated. Clinical information including vital signs, weight, body mass index (BMI), complete blood count, fasting plasma glucose (FBG), hemoglobin A1C, lipid profile, liver function test, total testosterone, estradiol, prolactin, follicular-stimulating hormone (FSH), luteinizing hormone (LH), 25-OH vitamin D, uric acid, blood urea nitrogen, creatinine, and thyroid function test, as collected both at baseline when CPA was initiated and at follow-up visits. Follow-up visits were at 3 months, 6 months, 9 months, 12 months, and then every 12 months.

For individuals AMAB who initiated CPA before receiving care at our institute, the times of CPA initiation were extracted and used for calculating interval times between the time of CPA initiation and the start of care at our institute. Then, the data were collected, if available, at 3 months, 6 months, 9 months, 12 months, and then every 12 months after the CPA initiation. Testosterone was measured using a chemiluminescent microparticle immunoassay (Alinity i) with an interassay coefficient of variation of 2.6% to 8.1% and a lower limit of quantification of 1.73 ng/dL. Medical records were carefully reviewed for any documented adverse effects, especially cardiovascular events, venous thromboembolism, hepatitis, depression, and meningioma.

### Outcome measures

The primary outcomes of interest included the median time to achieve testosterone suppression at the target level (<50 ng/dL) in the low-dose group (CPA <25 mg/day) and the standard-dose group (CPA 25-50 mg/day), the proportion of individuals AMAB who achieved the target testosterone level at 3 months, and the concentration of testosterone at each follow-up visit. Comparisons of prolactin, lipid profile, and liver enzyme concentrations were assessed between the 2 groups, along with cardiovascular events, venous thromboembolism, depression, and meningioma. Additionally, changes in other clinical data between the 2 groups were evaluated.

### Statistical analysis

The baseline characteristics were described using the mean and standard deviation or median and interquartile range (IQR), as appropriate for continuous variables and percentages for categorical variables. The differences between the low-dose and standard-dose groups were evaluated using the Ranksum, Mann–Whitney test for median comparison and Fisher’s exact test for categorical data. Only individuals AMAB who initiated CPA at the study site during the study period and had testosterone data at the 3-month follow-up were analyzed for the median time to achieve testosterone suppression at the target level and the proportion of individuals AMAB who achieved the target testosterone level at 3 months. A Cox regression analysis was used to compare the median time to achieve testosterone suppression at the target level, and a risk ratio regression model was used to compare the proportion of individuals AMAB who achieved the target testosterone level at 3 months between the 2 groups. Both univariable analysis and multivariable analysis adjusted for type of estrogen, smoking status, BMI, baseline testosterone level, baseline estradiol level, age at FHT initiation, age at CPA initiation, and use of other antiandrogens at CPA initiation were performed. The Cox regression analysis reported hazard ratios (HRs) for univariable analysis and adjusted HR (aHR) for multivariable analysis, and the risk ratio regression analysis reported relative risk (RR) and adjusted RR (aRR), respectively, with a significance threshold *P*-value <.05.

A multilevel mixed-effects linear regression was used to analyze testosterone level changes, presented as mean differences and 95% confidence intervals. The analysis was done for (1) individuals AMAB who initiated CPA during the study period at the study site and (2) an entire cohort, including individuals AMAB, who initiated CPA elsewhere before continuing care at the study site. Both non-adjusted analysis and adjusted analysis were performed. The analysis was adjusted for baseline testosterone levels, type of estrogen, smoking status, BMI, baseline estradiol level, age at FHT initiation, age at CPA initiation, and use of other antiandrogens at CPA initiation. Other repeated measures were analyzed with and without adjusted variables for each clinical data at baseline. A significance threshold for the *P*-value was <.05 (2-sided). Statistical analyses were performed using STATA version 18.0 (StataCorp®).

## Results

**Figure 1:**
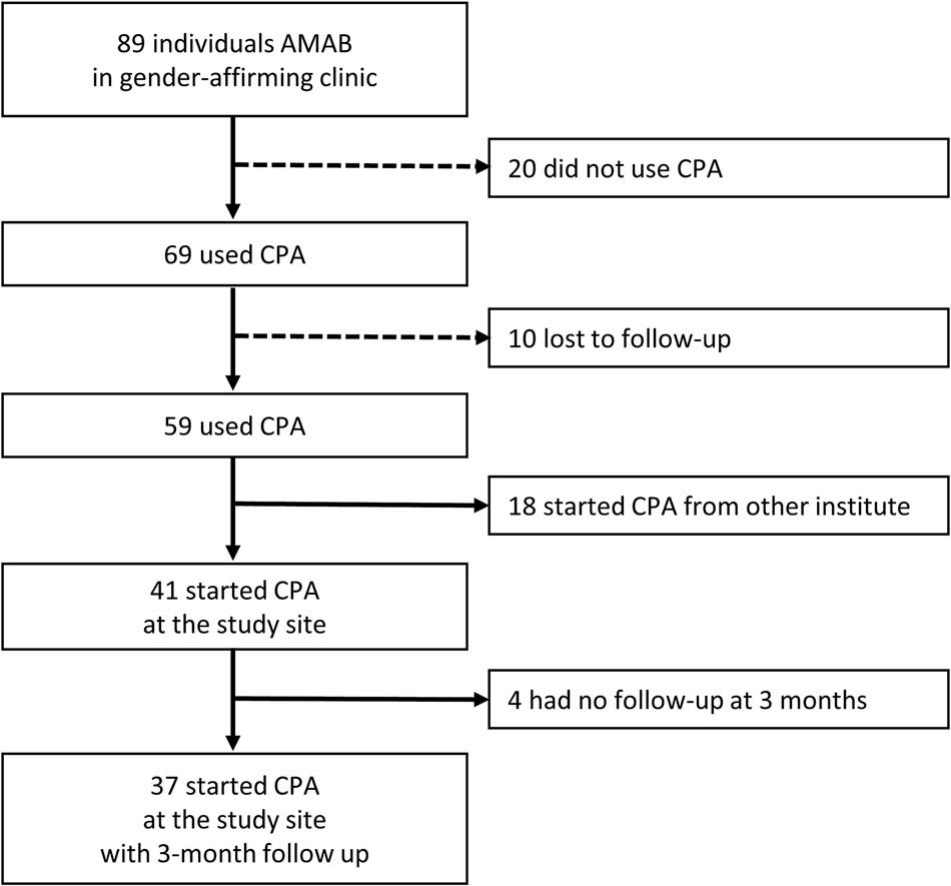
Study flow chart. Abbreviations: CPA = cyproterone acetate, AMAB = assigned male at birth.

A total of 89 individuals AMAB received FHT at the Gen V clinic. Sixty-nine subjects were prescribed CPA as their antiandrogen. Among these, 10 subjects were lost to follow-up, 18 had started CPA at another institute and relocated care to the study site, 4 initiated CPA at the study site but did not have a 3-month follow-up visit, and a total of 37 individuals AMAB initiated CPA with data at 3-month follow-up visit (Figure 1). Among 37 subjects, 22 started with CPA 12.5 mg/day (low-dose group), and 15 started with CPA 25–50 mg/day (standard-dose group). In the latter group, 14 individuals AMAB initiated with CPA at 25 mg/day, and only one started with CPA at 50 mg/day. The baseline characteristics of the 37 individuals AMAB who initiated CPA with available testosterone data at a 3-month follow-up visit are shown in Table 1. Overall, baseline characteristics were not significantly different between the 2 groups. The age of CPA initiation in the standard-dose group was slightly higher at 26 years, while the low-dose group was 20 years (*P* = .082). There were no differences in the baseline total testosterone levels, 517.5 ng/dL (IQR 173–787) in the low-dose group and 752.0 ng/dL (IQR 56–957) in the standard-dose group (*P* = .653). Five participants were self-prescribed FHT, including other anti-androgens: 3 out of 22 (13.6%) in the low-dose group and 2 out of 15 (13.3%) in the standard-dose group. Only LDL-C was statistically significantly different, with 128 mg/dL (IQR 116–136) in the low-dose group and 103 mg/dL (IQR 91–127) in the standard-dose group (*P* = .035). Additionally, there were no clinically relevant differences in BMI, systolic blood pressure (SBP), diastolic blood pressure (DBP), prolactin, estradiol, FBG, creatinine, liver enzymes, HDL-C, or triglycerides.

**Table 1:** Baseline characteristics of the 37 individuals AMAB who initiated CPA with available testosterone data at the 3-month follow-up visit.

	Low-dose CPA	Standard-dose CPA	*P*
Number (%)	22 (59.5)	15 (40.5)	
Age of FHT initiation, year (IQR)	19 (15–23)	20 (17–27)	.504
Age of CPA initiation, year (IQR)	20 (16–29)	26 (19–34)	.082
Follow-up time after using CPA, month (IQR)	7.5 (3–12)	9 (6–12)	.402
Use of other antiandrogen at the CPA initiation, *n* (%)	3 (13.64)	2 (13.33)	1
BMI, kg/m^2^ (ICR)	20.6 (19.6–26.4)	20.8 (19.1–22.2)	.867
Active smoking, *n* (%)	1 (4.6)	0	1
Testosterone, ng/dL (IQR)	517.5 (173–787)	752 (56–957)	.653
Estradiol, pg/mL (IQR)	30.2 (15.9–50.4)	31.1 (26.9–43.7)	.863
Prolactin, ng/mL (IQR)	14.1 (11.78–17.7)	15.36 (10.5–16.1)	.664
Lipid profile (IQR)			
LDL-C, mg/dL	128 (116–136)	103 (91–127)	.035^a^
HDL-C, mg/dL	55.5 (46–60)	60 (51–71)	.144
Triglyceride, mg/dL	84 (62–99)	71 (49–86)	.225
Liver enzymes, (IQR)			
AST, U/L	25 (20–27)	21.5 (21–28)	.836
ALT, U/L	22 (17–32)	19 (14–37)	.590
Creatinine, mg/dL (IQR)	0.78 (0.75–0.84)	0.81 (0.77–0.94)	.497
Fasting blood glucose, mg/dL (IQR)	92 (84–99)	88 (85–94)	.290
Systolic blood pressure, mmHg (IQR)	116 (111–124)	123.5 (117–130)	.102
Diastolic blood pressure, mmHg (IQR)	75 (72–79)	77.5 (76–83)	.194

a *P* < .05: significant differences between the 2 groups.

Abbreviations: AMAB = assigned male at birth, sFHT = feminizing hormone therapy, CPA = cyproterone acetate

**Figure 2:**
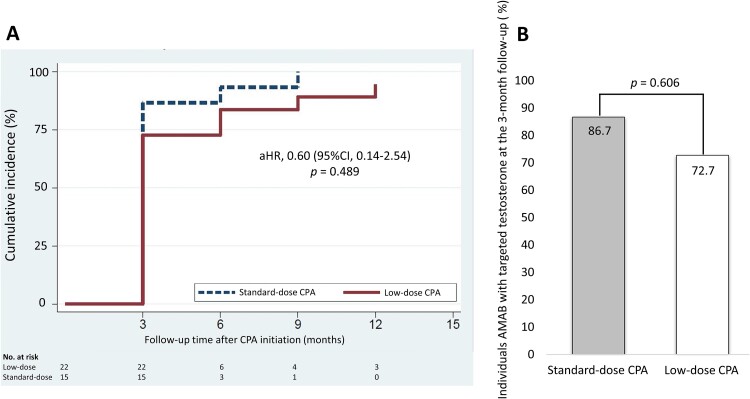
(A) Cumulative incidence of individuals AMAB who achieved targeted testosterone level (<50 ng/dL) after initiation of CPA. (B) The proportion of individuals AMAB who achieved targeted testosterone level at the 3-month follow-up after initiation of CPA. Abbreviations: CPA = cyproterone acetate, AMAB = assigned male at birth.

### Testosterone suppression

**Figure 3:**
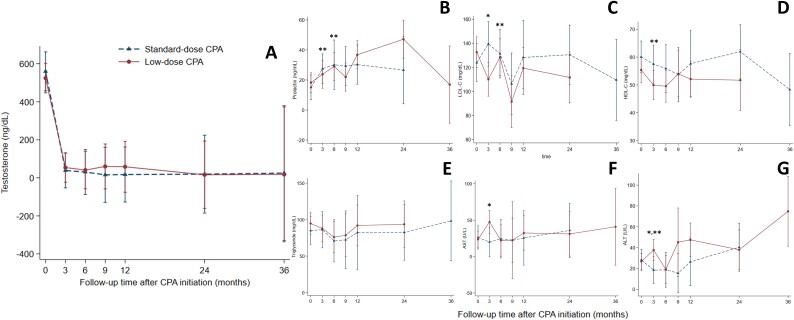
Trends of clinical data after initiation of cyproterone acetate at each follow-up visit. (A) Testosterone, (B) prolactin, (C) LDL-C, (D) HDL-C, (E) triglyceride, (F) AST, and (G) ALT. ^*^Significant differences between the 2 groups from non-adjusted analysis. ^*^^*^Significant differences between the 2 groups from adjusted analysis. Abbreviations: CPA = cyproterone acetate.

The median time to achieve testosterone suppression to the target level (<50 ng/dL) was 3 months in both groups (HR 0.79, 95% CI, 0.40–1.57, *P* = .505). A multivariable analysis for time to reach the targeted testosterone level for the low-dose group compared with the standard-dose group revealed no statistical difference (aHR 0.60, 95% CI, 0.14–2.54, *P* = .489). The cumulative incidence of achieving testosterone suppression is shown in Figure 2A. The proportion of individuals AMAB who achieved the targeted testosterone level within 3 months was 72.7% in the low-dose group and 86.7% in the standard-dose group, with RR 0.84 (95% CI, 0.60–1.17, *P* = .295) for univariable analysis. The multivariable analysis showed an aRR of 0.85 (95% CI, 0.46–1.57, *P* = .606) (Figure 2B). Testosterone levels at all follow-up visits and overall mean testosterone levels were not significantly different for both non-adjusted and adjusted repeated measure analyses between the groups. Testosterone levels at each visit among individuals AMAB who initiated CPA at our institution are shown in Figure 3A and Table 2. Moreover, the overall mean difference in testosterone levels in the entire cohort was not significant, as shown in Table S1. Only 1 individual AMAB in the low-dose group required a titration of CPA from 12.5 mg/day to 25 mg/day to achieve the targeted testosterone level, while none in the standard-dose group required such an adjustment.

### Prolactin, lipid profile, and liver enzymes

Prolactin, lipid profile, and liver enzymes in individuals AMAB who initiated CPA at our institution at each follow-up visit are shown in Figure 3 and Table 3. In the non-adjusted analysis, prolactin levels at each follow-up visit were not significantly different between the groups, with overall mean levels of 23.6 ng/mL (±3.1) in the standard dose and 25.8 ng/mL (±2.5) in the low dose group (Figure 3B). The standard-dose group had a non-significant lower mean prolactin level with a mean difference of −2.2 ng/mL (95% CI, −10.1 to 5.7, *P* = .581). An adjusted analysis showed that prolactin levels were significantly higher in a standard-dose group at 3-month and 6-month follow-up visits but not in the overall adjusted mean difference, which was 1.6 ng/mL (95% CI, −0.6 to 3.8, *P* = .152) (Table 3).

**Table 3:** Prolactin, lipid profile, and liver enzymes at each follow-up visit among 41 individuals AMAB who initiated CPA at our institution.

	Low-dose CPA		Standard-dose CPA		Non-adjusted analysis		Adjusted analysis^a^	
	Mean (SD)	*n*	Mean (SD)	*n*	Mean difference (95% CI)^b^	*P*	Mean difference (95% CI)^b^	*P*
Prolactin, ng/mL								
3 months	23.8 (4.8)	6	27.5 (5.1)	6	3.7 (−10.0, 17.3)	.600	11.9 (2.3, 21.6)	.015^c^
6 months	29.1 (4.7)	6	30.2 (8.4)	2	1.1 (−17.9, 20.0)	.913	12.8 (5.0, 20.6)	.001^c^
9 months	21.9 (4.8)	6	29.4 (6.6)	3	7.4 (−8.5, 23.4)	.361	6.6 (−1.3, 14.4)	.100
12 months	36.8 (4.8)	6	30.2 (6.7)	3	−6.5 (−22.6, 9.6)	.426	−5.6 (−15.0, 3.7)	.239
24 months	47.2 (6.5)	3	26.0 (−)	1	−20.6 (−46.4, 5.2)	.118	−6.9 (−16.3, 2.5)	.151
Overall	25.8 (2.5)		23.6 (3.1)		−2.2 (−10.1, 5.7)	.581	1.6 (−0.6, 3.8)	.152
LDL-C, mg/dL								
3 months	110.2 (7.2)	11	139.5 (9.4)	6	29.3 (6.1, 52.6)	.013^c^	6.7 (−10.7, 24.1)	.447
6 months	128.4 (7.8)	7	131.4 (10.3)	4	3.0 (−22.4, 28.4)	.818	−22.5 (−40.1, −4.8)	.013^c^
9 months	91.4 (11.0)	2	106.3 (13.1)	2	14.8 (−18.7, 48.4)	.386	−8.6 (−33.2, 16.0)	.492
12 months	119.4 (8.8)	5	128.0 (−)	1	8.9 (−26.4, 44.1)	.621	−7.6 (−29.4, 14.2)	.494
24 months	111.6 (10.8)	3	130.5 (12.5)	2	18.9 (−13.5, 51.4)	.253	0.7 (−22.4, 23.8)	.953
Overall	122.5 (6.2)		127.2 (7.4)		4.7 (−14.3, 23.7)	.626	−11.6 (−17.5, −5.7)	<.001^c^
HDL-C, mg/dL								
3 months	49.9 (2.7)	11	57.5 (3.5)	6	7.6 (−1.0, 16.2)	.085	8.6 (0.5, 16.7)	.037^c^
6 months	49.5 (2.9)	7	55.9 (4.4)	3	6.3 (−4.0, 16.7)	.229	9.3 (−0.1, 18.6)	.053
9 months	54.0 (4.2)	2	53.7 (5.0)	2	−0.3 (−13.1, 12.5)	.967	3.1 (−8.3, 14.6)	.592
12 months	52.1 (3.3)	5	57.6 (6.1)	2	5.6 (−8.0, 19.1)	.420	2.6 (−8.6, 13.8)	.646
24 months	51.7 (5.6)	2	61.0 (−)	1	10.3 (−4.4, 24.9)	.169	20.2 (1.4, 39.0)	.035
Overall	52.7 (2.2)		58.2 (2.7)		5.5 (−1.3, 12.3)	.114	6.2 (3.5, 8.9)	<.001^c^
Triglyceride, mg/dL								
3 months	87.9 (9.4)	11	86.1 (12.7)	6	−1.8 (−32.7, 29.2)	.911	8.3 (−17.2, 33.8)	.524
6 months	75.9 (10.9)	7	70.9 (14.7)	4	−5.1 (−41.1, 30.9)	.781	−4.4 (−31.2, 22.4)	.749
9 months	78.5 (15.0)	3	72.2 (20.2)	2	−6.3 (−55.5, 43.0)	.804	−10.2 (−45.9, 25.5)	.576
12 months	91.9 (14.3)	4	82.0 (−)	1	−9.7 (−68.0, 48.5)	.743	−6.3 (−50.2, 37.7)	.779
24 months	93.5 (16.3)	3	82.4 (19.5)	2	−11.1 (−61.0, 38.8)	.663	−7.0 (−53.2, 39.1)	.765
Overall	89.1 (6.8)		82.5 (8.2)		−6.6 (−27.5, 14.3)	.536	−2.1 (−11.7, 7.5)	.662
AST, U/L								
3 months	47.6 (8.1)	11	20.0 (10.2)	7	−27.6 (−53.1, −2)	.034^c^	−31.7 (−78.0, 14.6)	.179
6 months	22.1 (11)	6	24.4 (13.5)	4	2.4 (−31.8, 36.5)	.893	−2.1 (−65.8, 61.7)	.949
9 months	22.0 (−)	1	22.3 (15.6)	3	−0.2 (−61.3, 60.8)	.994		
12 months	32.4 (12.1)	5	25.5 (19.1)	2	−6.9 (−51.1, 37.3)	.761	−5.6 (−70.4, 59.2)	.865
24 months	31.1 (15.6)	3	35.9 (19.1)	2	4.8 (−43.4, 53.1)	.844	3.1 (−91.2, 97.3)	.949
Overall	30.6 (3.9)		24.7 (5.1)		−5.9 (−18.5, 6.7)	.358	−6.2 (−20.5, 8.1)	.393
ALT, U/L								
3 months	37.6 (5.2)	11	18.3 (6.5)	7	−19.3 (−35.6, −2.9)	.021^c^	−24.9 (−47.2, −2.6)	.028^c^
6 months	19.0 (6.8)	6	18.6 (8.5)	4	−0.3 (−21.8, 21.1)	.975	6.1 (−33.4, 21.1)	.658
9 months	45.0 (−)	1	15.4 (9.6)	3	−29.7 (−67.7, 8.2)	.125		
12 months	47.4 (8.2)	5	26.3 (11.7)	2	−21.1 (−49.1, 6.9)	.139	−19.9 (−52.6, 12.8)	.233
24 months	37.9 (9.7)	3	40.3 (11.8)	2	2.4 (−27.6, 32.3)	.876	1.8 (−44.5, 48.2)	.938
Overall	32.1 (3.2)		24.0 (3.9)		−8.1 (−17.9, 1.7)	.106	−8.4 (−15.4, −1.4)	.019^c^

a Adjusted for baseline levels of each parameter

b Difference in clinical parameters in the standard-dose group compared with the low-dose group.

c *P* < .05: significant differences between the 2 groups.

Abbreviations: AMAB = assigned male at birth, CPA = cyproterone acetate

A non-adjusted analysis showed that LDL-C levels were higher in the standard-dose group only at the 3-month follow-up, with mean levels of 139.5 mg/dL (±9.4) compared to 110.2 mg/dL (±7.2) in the low-dose group, resulting in a mean difference of 29.3 mg/dL (95% CI, 6.1–52.6, *P* = .013). LDL-C levels were not different in other visits and in the overall mean levels (Figure 3C). An adjusted analysis revealed significantly lower overall mean LDL-C levels in the standard-dose group, with an adjusted mean difference of −11.6 mg/dL (95% CI, −17.5 to −5.7, *P* = <.001) and at the 6-month follow-up visit (Table 3). HDL-C levels were not different at all visits and in the overall mean levels in the non-adjusted analysis, with a mean difference of 5.5 (95% CI, −1.3 to 12.3, *P* = .114) (Figure 3D). An adjusted analysis revealed a significantly higher HDL-C level in the standard-dose group at the 3-month follow-up and for the overall adjusted mean difference of 6.2 (95%CI, 3.5–8.9, *P* = <.001) (Table 3). Triglyceride levels did not differ between the groups at all visits, with the overall mean difference for both non-adjusted and adjusted analyses (Figure 3E and Table 3).

**Table 2:** Testosterone levels (ng/dL) at each follow-up visit among 41 individuals AMAB who initiated CPA at our institution.

	Low-dose CPA *n* = 25		Standard-dose CPA *n* = 16		Non-adjusted analysis		Adjusted analysis^a^	
	Mean (SD)	*n*	Mean (SD)	*n*	Mean difference (95% CI)^b^	*P*	Mean difference (95% CI)^b^	*P*
3 months	54.0 (39.5)	22	38.5 (46.7)	15	−15.5 (−135.4, 104.4)	.800	−19.9 (−67.3, 27.5)	.410
6 months	40.8 (50.2)	13	30.3 (60.3)	9	−10.5 (−164.2, 143.3)	.894	−19.3 (−76.4, 37.8)	.507
9 months	59.7 (60.3)	9	15.7 (73.9)	6	−44.1 (−231, 142.9)	.644	−50.6 (−114.3, 12.9)	.119
12 months	58.1 (68.4)	7	17.3 (73.9)	6	−40.8 (−238.1, 156.5)	.685	−39.4 (−123.2, 44.4)	.357
Overall	180.3 (20.8)		151 (25.1)		−29.3 (−93.1, 34.5)	.368	−32 (−96.3, 32.3)	.330

a Adjusted for baseline testosterone, type of estrogen, smoking status, BMI, baseline estradiol, age at FHT initiation, age at CPA initiation, and use of other antiandrogens at CPA initiation.

b Difference in testosterone levels in the standard-dose group compared with the low-dose group.

Abbreviations: AMAB = assigned male at birth, CPA = cyproterone acetate

Regarding liver enzymes, a non-adjusted analysis showed lower aspartate aminotransferase (AST) levels in the standard-dose group only at the 3-month follow-up (Table 3), but AST levels were not different at other visits or in overall mean levels, with a mean difference of −5.9 U/L (95% CI, −18.5 to 6.7, *P* = .358) (Figure 3F). An adjusted analysis showed no difference in AST levels at all follow-up visits and in the overall adjusted mean difference. ALT levels were also lower in the standard-dose group only at the 3-month follow-up in the non-adjusted analysis (Table 3). ALT levels were not different at other visits or in overall mean levels, with a mean difference of −8.1 U/L (95% CI, −17.9 to 1.7, *P* = .106) (Figure 3G). An adjusted analysis showed significantly lower ALT levels in the standard-dose group at the 3-month follow-up visit and an overall adjusted mean difference of −8.4 U/L (95% CI, −15.4 to −1.4, *P* = .019) (Table 3). Among the entire cohort, overall mean differences in prolactin, LDL-C, HDL-C, triglyceride, AST, and ALT were not different (Table S1).

### Other clinical parameters

A non-adjusted analysis of overall mean differences in other clinical parameters, including estradiol, fasting blood sugar, creatinine, 25-hydroxy vitamin D, SBP, DBP, and BMI, was not statistically different between the 2 groups in individuals AMAB who initiated CPA at our institution (Table S2). DBP at the 3-month follow-up was higher in the standard-dose group, with a mean difference of 5.7 mmHg (95% CI, 1.0–10.4, *P* = .018). Other parameters were not different at all follow-up visits.

An adjusted analysis reiterated the higher DBP in the standard-dose group at the 3-month follow-up (Table S2) with an adjusted mean difference of 5.5 mmHg (95% CI, 1.2–9.9, *P* = .012). Overall mean fasting blood glucose was lower in the standard-dose group by −25.9 mg/dL (95% CI, −35 to −16.8, *P* = <.001). Moreover, BMIs were lower in the standard-dose group at all follow-up visits, including an overall adjusted mean difference of −1.5 kg/m^2^ (95% CI, −2.1 to −1.0, *P* = <.001) (Table S2). In the entire cohort, overall mean differences of all clinical parameters were not statistically different. There was insufficient data on A1C, FSH, LH, uric acid, and thyroid-stimulating hormone (TSH) for analysis.

### Adverse outcomes

None of the participants were diagnosed with myocardial infarction, cerebrovascular accident, venous thromboembolism, new-onset depression, or meningioma during the study period.

## Discussion

The present study shows that initiating a low-dose CPA with the dose of 12.5 mg/day was able to achieve targeted testosterone levels with a median time of 3 months, not significantly different from the standard-dose CPA of 25–50 mg/day. Although testosterone levels were not statistically different at baseline, some experts may argue that the levels of 517.5 ng/dL in the low-dose group and 752.0 ng/dL in the standard-dose group represent clinically significant differences. Despite the higher baseline testosterone level in the standard-dose group, testosterone levels were comparable during follow-up visits between the low-dose and standard-dose CPA groups. Additionally, the proportion of individuals AMAB who achieved the targeted testosterone level at the first follow-up visit at 3 months was similar between the 2 groups. Regarding other clinical parameters, the standard-dose CPA group showed overall lower LDL-C, higher HDL-C, and lower ALT during the follow-up period. Among the entire cohort, including individuals AMAB who initiated CPA before receiving care at our institute, the overall mean differences in all clinical data were not significant.

The most recent SOC8 from the WPATH recommends a CPA dose of less than 10 mg/day.^1^ This recommendation is primarily based on concerns about the risk of meningioma associated with higher doses of CPA in both transgender and cisgender individuals. A French cohort study of cisgender women revealed a strong dose-effect relationship between CPA and meningiomas. Participants exposed to CPA had a 6.6-fold higher risk compared to the control group, with the risk increasing up to 21-fold in high-dose users (cumulative dose greater than 60 g).^18^ A retrospective study from the Netherlands involving 2555 individuals AMAB using CPA 50–100 mg/day revealed a higher standardized incidence ratio of meningioma than in a general European population.^19^ The incidence was 4.1 times higher compared to the female population and 11.9 times higher than the male population. Notably, most individuals AMAB used CPA despite having undergone orchiectomy. A summary of 8 meningioma cases in individuals AMAB using CPA reported in the literature revealed that 7 of them used 50–100 mg/day, and one individual AMAB used a low dose of 10 mg/day.^20^ Although no meningioma cases were reported in the present cohort, the follow-up time was relatively short. Finally, the European Medicines Agency’s safety committee launched a recommendation in 2020 that CPA doses higher than 10 mg/day should be restricted.^21^ Notably, the SOC8 does not provide information regarding the effectiveness of the recommended lower CPA dose in testosterone suppression.

A few published studies have shown promising results in testosterone suppression using low-dose CPA as feminizing hormone therapy. The European Network for the Investigation of Gender Incongruence (ENIGI), a multicenter prospective cohort study, demonstrated that a CPA dose of 10 mg/day was comparable to higher doses (25, 50, and 100 mg/day) in terms of testosterone suppression at both 3 and 12 months of hormone therapy.^22^ However, it is important to note that only 4 individuals AMAB were in the 10 mg CPA group. A historical cohort study from Israel revealed similar testosterone suppression at 6 months of hormone therapy between 38 individuals AMAB using low-dose CPA (10–20 mg/day) and 26 individuals AMAB using CPA 50–100 mg/day.^23^ Despite the larger sample size, this study did not report data at the 3-month mark. The present study reiterates the effectiveness of low-dose CPA (12.5 mg/day) in testosterone suppression and provides data at the 3-month follow-up after hormone therapy. A recent Australian study utilized a titration protocol to determine the lowest effective dose of CPA. For CPA-naïve individuals, the protocol began with 12.5 mg of CPA daily and 25 mg on alternate days. The CPA dose was gradually reduced to maintain target testosterone levels. The study found that 69% of participants could suppress testosterone with a dose of 12.5 mg daily/25 mg on alternate days or lower. Out of 19 individuals on the lowest CPA protocol (12.5 mg twice a week), 57.9% (11/19) achieved their testosterone goals.^24^

Hyperprolactinemia is one of the concerning adverse effects of CPA, yet its clinical significance remains unclear. Initial data from a study in Belgium indicated that prolactin elevation resolved after discontinuing CPA in individuals AMAB who had undergone orchiectomy^25^ and continued solely with estrogen. This finding suggests that the elevated prolactin level was primarily due to CPA, not estrogen. A systematic review of 17 studies showed that prolactin levels increased by 152–297% with CPA use.^26^ However, the risk of prolactinomas remains equivocal. The present study revealed higher prolactin levels in the standard-dose CPA group during early follow-up visits (3 months and 6 months) but not in an overall means for the entire follow-up period. Both the ENIGI study and the study from Israel, which compared low-dose and high-dose CPA, demonstrated a dose-dependent relationship between CPA dose and prolactin elevation.^22^^,^^23^ Furthermore, the rise in prolactin levels did not reach the range of hyperprolactinemia at any CPA dose in the ENIGI study.^22^

A negative effect of CPA on lipid profile has been reported in the literature. A retrospective cohort of 126 individuals AMAB comparing different anti-androgens revealed that CPA resulted in lower HDL-C compared to spironolactone and GnRH agonists.^27^ A recent meta-analysis demonstrated that feminizing hormone therapy using progestogens, including CPA, showed a significant decrease in HDL-C.^28^ The ENIGI study, comparing several doses of CPA, revealed the dose-dependent HDL-C lowering effect. However, HDL-C levels in the 10 mg CPA group were not different from those in the group not using CPA.^22^ Previous data indicated that the effect of CPA on LDL-C and triglyceride was neutral. Surprisingly, the present study showed a significantly higher HDL-C level and lower LDL-C in the standard-dose group. However, these results may not be clinically significant.

CPA is not approved in the U.S. due to concerns about potential hepatotoxicity. Data regarding hepatotoxicity from CPA are mainly based on case reports, with hepatitis being the most common type of liver injury.^29^ The ENIGI study revealed lower AST levels in individuals AMAB using CPA compared to those not using CPA. ALT levels in CPA doses of 10 mg, 25 mg, 50 mg, and 100 mg were not significantly different, and there were no relevant differences in AST between groups.^22^ A study from Israel reported hepatitis in 3 out of 26 individuals AMAB using high-dose CPA (50–100 mg), while there were no cases in the low-dose CPA (10–20 mg) group.^23^ Our study found that ALT levels were slightly lower in the standard-dose group; however, the clinical significance of this finding is questionable.

The present study is one of the few that demonstrates the effectiveness of low-dose CPA in testosterone suppression, aligning with the current paradigm of FHT. This study includes the largest cohort of individuals AMAB at the 3-month follow-up using low-dose CPA, compared to only 4 individuals AMAB in the 10 mg CPA group in the ENIGI study.^18^ This study includes the largest cohort of individuals AMAB at the 3-month follow-up using low-dose CPA, compared to only 4 individuals AMAB in the 10 mg CPA group in the ENIGI study.^19^ However, several limitations exist. First, the sample sizes were small, and there was significant missing data on clinical parameters other than testosterone levels due to the retrospective nature of the study. Additionally, no sample size calculation was performed, and the smaller number of participants in later follow-up visits may not provide enough power to demonstrate significant differences. The authors acknowledge this limitation and present data on other clinical parameters as supplementary information, as they are not the primary focus of this study. Furthermore, the follow-up period was short and may not have been long enough to observe adverse outcomes, particularly meningiomas or cardiovascular events.

A trend in using CPA in FHT is moving toward lower doses. Future research should focus on the efficacy and adverse effects of low-dose CPA in testosterone suppression with well-controlled studies. CPA has a half-life elimination of approximately 48 ho.^30^ This characteristic suggests the potential for less than daily dosing of CPA in clinical practice. Therefore, future studies should investigate the efficacy and safety of CPA at doses less than 10 mg/day or non-daily dosing regimens.

## Conclusion

In conclusion, the present study demonstrated that low-dose CPA (12.5 mg/day) in FHT yielded comparable effectiveness in testosterone suppression to standard-dose CPA (25-50 mg/day) as early as 3 months after hormone initiation. The standard dose was associated with higher HDL-C but lower LDL-C, ALT, fasting blood glucose, and BMI. However, the clinical significance of these differences is questionable. This study supports the recommendation of using a lower dose of CPA in individuals AMAB in terms of effectiveness in lowering testosterone levels.

## Supplementary Material

Supplementary_tables_qfae063

## Data Availability

The data sets from the present study are not publicly available.
