# Supplementary material for: Effectiveness of low dose cyproterone acetate compared to standard dose in feminizing hormone treatment: a single institutional retrospective pilot study
Source: Sex Med. 2024 Sep 21;12(4):qfae063. doi: 10.1093/sexmed/qfae063 (PMC11416909; doi:10.1093/sexmed/qfae063)
Supplement: Supplementary_tables_qfae063 [file supplementary_tables_qfae063.pdf]

Table S1. Clinical data at each follow-up visit among 41 individuals AMAB who initiated CPA at our institution.

	Low-dose CPA ,mean (SD)	Standard-dose CPA, mean (SD)	Non-adjusted analysis		Adjusted analysis[1]	
			Mean difference (95% CI)[2]	*p*	Mean difference (95% CI)[2]	*p*
Estradiol, pg/mL						
Baseline	44.2 (18.1)	37.1 (24.3)	-7.1 (-66.5, 52.3)	0.815	-	-
3 months	67.8 (18.5)	61.3 (22.1)	-6.5 (-62.9, 49.9)	0.821	-17.4 (-87.1, 52.3)	0.625
6 months	87.4 (21.4)	69.9 (27.6)	-17.5 (-86.0, 50.9)	0.615	-31.1 (-112.1, 49.9)	0.452
9 months	75.3 (25.6)	90.3 (30.8)	15.1 (-63.5, 93.6)	0.707	2.3 (-96.8, 101.4)	0.964
12 months	74.9 (25.7)	84.5 (30.9)	9.6 (-69.1, 88.3)	0.811	0.3 (-99.1, 99.7)	0.995
24 months	79.6 (35.3)	80.5 (39.7)	0.9 (-103.3, 105.1)	0.986	2.7 (-125.5, 130.8)	0.967
Overall	67.7 (12.7)	66.0 (15.8)	-1.8 (-41.5, 38.0)	0.931	-11.5 (-45.3, 22.3)	0.506
Fasting blood glucose, mg/dL						
Baseline	100.3 (8.3)	87.8 (12.4)	-12.6 (-41.8, 16.7)	0.400	-	-
3 months	121.6 (14.9)	94.3 (17.5)	-27.3 (-72.5, 17.8)	0.236	-	-
6 months	102.7 (25.5)	68 (35.1)	-34.7 (-119.7, 50.3)	0.424	-	-
9 months	-	88 (24.8)	-	-	-	-
12 months	80.8 (12.7)	88.7 (26.4)	7.9 (-49.5, 65.4)	0.787	-	-
24 months	90 (23.7)	92 (35.1)	2 (-80.9, 84.9)	0.962	-	-
Overall	100.3 (7.7)	88.5 (8.8)	-11.8 (-34.7, 11.1)	0.311	-25.9 (-35, -16.8)	<0.001*
Creatinine, mg/dL						
Baseline	0.8 (0.03)	0.8 (0.03)	0.05 (-0.04, 0.13)	0.293	-	-
3 months	-	0.9 (0.06)	-	-	-	-
6 months	-	0.8 (0.05)	-	-	-	-
9 months	0.7 (0.12)	0.7 (0.05)	0.06 (-0.19, 0.31)	0.656	-	-
12 months	0.8 (0.04)	1 (0.08)	0.20 (0.03, 0.38)	0.022*	-	-
24 months	0.8 (0.06)	0.9 (0.08)	0.14 (-0.06, 0.33)	0.163	-	-
Overall	0.8 (0.03)	0.8 (0.03)	0.06 (-0.02, 0.13)	0.142	0.1 (0.0, 0.1)	1.000
25-hydroxy vitamin D, ng/mL						
Baseline	15.5 (2.5)	17.0 (6.8)	1.4 (-12.8, 15.6)	0.843	-	-
3 months	26.5 (3)	15.4 (9.6)	-11.1 (-30.7, 8.6)	0.27	-11.1 (-30.7, 8.6)	0.27
6 months	38.2 (3.8)	43.5 (9.6)	5.3 (-14.9, 25.5)	0.606	5.3 (-14.9, 25.5)	0.606
9 months	24.4 (5.4)	36.4 (9.6)	12.0 (-9.6, 33.6)	0.276	12.0 (-9.6, 33.6)	0.276
12 months	25.7 (4.2)	23.3 (9.6)	-2.4 (-22.8, 18.1)	0.821	-2.4 (-22.8, 18.1)	0.821
24 months	22.6 (6.6)	39.2 (9.6)	16.6 (-6.1, 39.4)	0.152	16.6 (-6.1, 39.4)	0.152
Overall	24.0 (1.7)	27.4 (3.7)	3.4 (-4.6, 11.4)	0.406	3.4 (-4.6, 11.4)	0.406
Systolic blood pressure, mmHg						
Baseline	118.2 (2.2)	121.4 (2.7)	3.2 (-3.6, 10.1)	0.353	-	-
3 months	117.8 (2.4)	124 (2.7)	6.2 (-0.9, 13.4)	0.089	5.7 (-0.9, 12.3)	0.090*
6 months	114.2 (3.2)	113.4 (3.5)	-0.8 (-10, 8.4)	0.866	-1.5 (-10.0, 7.1)	0.738
9 months	121.1 (3.5)	117.4 (4.2)	-3.7 (-14.4, 7)	0.500	-4.6 (-14.7, 5.5)	0.371
12 months	119.1 (3.5)	119.5 (6.3)	0.5 (-13.6, 14.5)	0.947	-0.3 (-13.8, 13.2)	0.969
24 months	128.7 (6.3)	-	-		-	-
Overall	118.3 (1.8)	120.3 (2.2)	2.1 (-3.5, 7.7)	0.471	1.1 (-4.0, 6.2)	0.677
Diastolic blood pressure, mmHg						
Baseline	76.2 (1.4)	78.6 (1.8)	2.3 (-2.1, 6.8)	0.308	-	-
3 months	74.4 (1.6)	80.1 (1.8)	5.7 (1.0, 10.4)	0.018*	5.5 (1.2, 9.9)	0.012*

6 months	74.1 (2.0)	70.9 (2.2)	-3.2 (-9.1, 2.7)	0.291	-3.5 (-9.0, 2.0)	0.216
9 months	75.2 (2.2)	77.6 (2.7)	2.3 (-4.5, 9.2)	0.507	2.1 (-4.5, 8.6)	0.534
12 months	78.4 (2.2)	76.7 (4)	-1.7 (-10.6, 7.2)	0.712	-1.7 (-10.6, 7.2)	0.704
24 months	85.6 (4.0)	-	-	-	-	-
Overall	76.0 (1.2)	77.5 (1.5)	1.4 (-2.3, 5.2)	0.448	1.0 (-1.2, 3.2)	0.364
Body mass index, kg/m^2						
Baseline	22.9 (1.0)	23.3 (1.2)	0.4 (-2.6, 3.5)	0.793	-	-
3 months	22.6 (1.0)	22.4 (1.2)	-0.2 (-3.3, 2.8)	0.886	-2.2 (-3.4, -1.1)	<0.001*
6 months	22.9 (1.0)	22.9 (1.3)	0.0 (-3.2, 3.2)	0.993	-2.1 (-3.3, -0.9)	<0.001*
9 months	23.8 (1.0)	22.9 (1.3)	-0.9 (-4.1, 2.4)	0.600	-2.9 (-4.2, -1.6)	<0.001*
12 months	23.8 (1.0)	23.2 (1.4)	-0.5 (-4, 2.9)	0.761	-2.9 (-4.3, -1.5)	<0.001*
24 months	25.0 (1.3)	-	-	-	-	-
Overall	23.1 (1.0)	23.0 (1.2)	-0.1 (-3.2, 2.9)	0.925	-1.5 (-2.1, -1.0)	<0.001*

[1]Adjusted for baseline levels of each parameter

[2]Difference in clinical parameters in the standard-dose group compared with the low-dose group.

*p<0.05: significant differences between the two groups.

Abbreviations: AMAB = assigned male at birth, CPA = cyproterone acetate

Table S2. Overall mean differences of clinical parameters among an entire cohort, 57 individuals AMAB

	Low-dose CPA, mean (SD)	Standard-dose CPA, mean (SD)	Mean difference (95% CI)[1]	*p*
Testosterone, ng/dL	160.8 (17.5)	138.4 (21.8)	-22.4 (-77.2, 32.5)	0.424
Prolactin, ng/mL	24.8 (2.0)	24.5 (2.6)	-0.3 (-6.7, 6.2)	0.938
LDL-C, mg/dL	126.8 (4.7)	127.9 (6.0)	-1.2 (-13.8, 16.2)	0.879
HDL-C, mg/dL	53.5 (1.7)	56.1 (2.1)	2.6 (-2.7, 7.8)	0.339
Triglyceride, mg/dL	83.2 (5.0)	78.1 (6.3)	-5.0 (-20.9, 10.8)	0.532
AST, U/L	28.2 (3.0)	24.4 (3.9)	-3.8 (-13.5, 5.9)	0.441
ALT, U/L	28.0 (2.5)	24.6 (3.3)	-3.4 (-11.5, 4.7)	0.407
Estradiol, pg/mL	62.9 (9.8)	62.7 (12.6)	-1.8 (-41.5, 38.0)	0.931
Fasting blood glucose, mg/dL	97.9 (5.4)	88.5 (6.8)	-9.4 (-26.3, 7.6)	0.279
Creatinine, mg/dL	0.79 (0.02)	0.82 (0.26)	0.31 (-0.33, 0.95)	0.338
25-hydroxy vitamin D, ng/mL	24.5 (1.4)	23.6 (2.7)	-0.9 (-6.9, 5.0)	0.762
Systolic blood pressure, mmHg	118.3 (1.4)	121.0 (1.8)	2.7 (-1.7, 7.1)	0.223
Diastolic blood pressure, mmHg	76.0 (1.0)	78.6 (1.2)	2.6 (-0.4, 5.7)	0.092
Body mass index, kg/m^2	23.0 (0.9)	24.3 (1.2)	1.3 (-1.7, 4.4)	0.385

[1]Difference in clinical parameters in the standard-dose group compared with the low-dose group.

Abbreviations: AMAB = assigned male at birth, CPA = cyproterone acetate
